# Examining the roles of partnerships in enhancing the health systems response to COVID-19 in Nigeria

**DOI:** 10.1186/s12913-023-09827-4

**Published:** 2023-08-14

**Authors:** Chioma Amarachi Onyedinma, Chinyere Cecilia Okeke, Obinna Onwujekwe

**Affiliations:** 1 Health Policy Research Group, College of Medicine, University of Nigeria, Nsukka, Nigeria; 2 Department of Community Medicine, University of Nigeria Teaching Hospital, Ituku/Ozalla, Enugu, Nigeria; 3 Department of Health Administration and Management, Faculty of Health Sciences and Technology, University of Nigeria Enugu, Nsukka, Nigeria

**Keywords:** Partnerships, Health system response, COVID-19

## Abstract

**Background:**

The COVID-19 pandemic overwhelmed the health systems and socio-economic foundations of many countries, Nigeria inclusive. The study was carried out to assess, understand, document and report the activities/measures that are considered nationally and sub-nationally significant, both in terms of COVID-19 responses and in terms of strengthening the health system for the future, in response to future threats since this will not be the last pandemic This paper examines how partnerships contributed to the health system and other sectors’ responses to COVID − 19 infection in Nigeria.

**Methods:**

This was a qualitative study. Data was collected using a scoping literature review and key informant interviews with 36 key stakeholders in the COVID-19 response in Nigeria, in Abuja (national level) Lagos and Enugu states (sub-national level). Interviews were recorded and transcribed verbatim. The qualitative data was analysed using thematic analysis.

**Results:**

It was found that many partnerships were formed when responding to the COVID-19 pandemic in Nigeria. The health system leaned towards a horizontal dimension of partnership with non-health governmental sectors, non-governmental sectors, and other countries. All the components of the health system building blocks had a measure of partnership contributing to its accomplishments The partnerships came in varied forms, ranging from advocacy, funding, provision of palliatives to the citizens because of lockdowns, technical assistance, support to research, development of guidelines and health educational materials.

**Conclusion:**

The health sector’s collaboration with other sectors strengthened all the building blocks of the health system and was invaluable in enhancing the response to COVID-19, which needed a whole of government and a multi-sectoral approach. Formal frameworks for quickly initiating whole-of-government and multi-sectoral partnerships should be developed, with clear roles and responsibilities. This should be deployed for health system resilience and for response to shocks such as the COVID-19 pandemic.

## Background

The COVID-19 outbreak is one of the most widely spread infections of the respiratory tract that caused many mortalities, affecting so many countries at the same time. The weak health system of most low- and middle-income countries (LMICs), Nigeria inclusive, made the country very vulnerable to this outbreak. According to the World Health Organization (WHO), Nigeria was classified as one of the 13 high-risk African countries in relation to the outbreak [1].

From the beginning of the pandemic, the Federal Ministry of Health (FMOH), although saddled with the responsibility of overseeing the health issues of the country, did not embark on the mission to fight the pandemic on its own. The country formed a Coronavirus Preparedness Group with representation from varied organizations like the Office of the National Security Adviser (ONSA), Public Health England (PHE), WHO among others, and the Nigerian Centre for Disease Control (NCDC) which is the agency responsible for controlling diseases outbreaks in Nigeria [2]. The Presidential Task Force PTF, which is now the Presidential Steering Committee (PSC) that was set up at the time also had representation from the ministries of health, foreign affairs, humanitarian affairs and information as well as the NCDC to mention but a few to coordinate a multi-stakeholder response to the pandemic [3].

The multi-stakeholder response to the pandemic was also adopted by other countries such as Jamaica, Timor-Lest [4, 5]. This could have been informed by one of the principles of primary healthcare, which is intersectoral collaboration, simply defined as the coming together of different people, organizations and sectors to work together to understand and solve complex issues [6]. In the context of primary healthcare, it is a population approach to healthcare and health outcomes and can take different forms like alliances, cooperative initiatives, coalitions, or partnerships [7]. This collaboration or partnership can take a horizontal or vertical dimension. The vertical dimension is partnership at different levels of a given sector for example, collaboration with frontline health workers and health policy makers in an organization while the horizontal form links the health sector with different sectors, and this could be other governmental sectors like security, finance environment and then non-governmental organizations like the private, not for profit and voluntary organizations [7].

Health partnerships are a blueprint for boosting health and health services based on synergy and shared ideas between actors and institutions from different countries [8]. Hence, partnerships with other sectors are also quite important, considering the social determinants of health and the current global priority of mainstreaming health in all sectors, using the Health-in-all-policies concept. According to Abubakar et al., while a well-functioning and resourced health system will be vital to improve the health of Nigerians, much of the disease burden experienced across the country results from factors that lie outside the health system. A multi-sectoral or ‘Health-in-all-Policies (HiaP)’ approach to address key risk factors and social determinants of health including nutrition, access to clean water and sanitation, family planning and healthy environments would be a cost-effective approach to improve population health outcomes and drive sustainable development, while simultaneously relieving pressure on health services [9].

The Nigerian health sector, in managing the COVID-19 crises adopted the horizontal approach of partnership that linked the health sector with other sectors including the private sector, bilateral and multilateral organizations [3, 10, 11]. Partnership, (public private partnership) has helped to improve health service in Nigeria [12]. The public private partnership that exists between Nigeria’s tertiary health facility with private organizations in running their diagnostic centres has reduced the waiting time for patients accessing diagnostic care, provided state of the art equipment with better diagnostic capacities and has reduced result generation time [12]. However, many public private partnership that exist in Nigeria last for a while but are not permanent and are based on a concept of reciprocal learning and mutual benefit [8].

In the context of our study, the Nigerian health system response to COVID-19 was described using a framework adapted from the WHO building block framework for health system. The building blocks describe the health system in terms of six components: service delivery, health workforce, health information systems, access to essential medicines, healthcare financing, and leadership and governance. The study was carried out to assess, understand, document and report the activities/measures that are considered nationally and sub-nationally significant, both in terms of COVID-19 responses and in terms of strengthening the health system for the future, in response to future threats since this will not be the last pandemic.

This paper describes how partnerships contributed to the accomplishments noted in the country’s COVID − 19 health system response, as well as the dimension and the forms of partnerships that existed.

## Methods

This paper is a component of a study titled: Exploring and learning from regional, national and sub-national evidence, policy and systems responses to COVID-19 in West and Central Africa, a project that sought to build collaborative learning in and across countries and regionally in West Africa and Central Africa (DRC) between formal reported evidence and the experiential learning from implementation in countries.

Nigeria is the most populous country in Africa with a population of over 170 million people, accounting for 50% of West Africa’s population [13]. The country is made up of about 200 ethnic groups, 500 indigenous languages, and two major religions - Islam and Christianity [13]. The significant federal government representation on ethnic and cultural identities creates a balance for the wide variation in the geographical, ethnic and cultural identities [13].

Both the public and private sector provide healthcare services in Nigeria and Nigeria operates a 3-tierd system of government, the federal (national level), state, and local governments. The Public healthcare services functions at the 3 tiers of government. The federal government, State government and Local government are primarily responsible for the tertiary, secondary and primary levels of care respectively [14]. There are several health agencies at the 3 levels of government some of these health agencies like the Nigerian Centre for Disease Control (NCDC), Nigerian Institute of Medical Research (NIMR), National Agency for Food and Drug Administration (NAFDAC), National Institute for Pharmaceutical Research and Development (NIPRD), National Primary Health Care Development Agency (NPHCDA) to mention but a few have been playing vital roles in the control of the COVID-19 pandemic.

The study was carried out at the national and subnational levels in Nigeria. At the national level, the study was carried out at the Federal capital territory Abuja while at the sub-national level, the study was carried out in Lagos and Enugu States. Lagos was chosen because it was the epicentre of the disease in Nigeria, with a lot of activities and key players at various levels, while Enugu State was chosen by convenience.

### Study design

This was a qualitative study. We conducted a scoping literature review and key informant interviews. One hundred and ninety-eight documents were reviewed. The review included published government documents on COVID-19, media articles, and peer-reviewed and non-peer-reviewed publications describing the COVID-19 health system response in general. We used key words such as COVID-19; Coronavirus; Nigeria; Lagos; Enugu; Health system; health sector; using terms such as Health systems; Strengthening; etc. as search terms in the following search engines; PubMed, Google, Google Scholar and FACTIVA. The government websites searched were FMOH, NCDC, NCAA, SMOHs’ NAFDAC, NCC NIMR, NIPRD etc. and other websites searched include the World Bank, WHO-Afro and John Hopkins School of Public Health.

Thirty-six key informant interviews (KIIs) were conducted to further understand and validate country’s COVID-19 response approaches, practices, and strategies. we conducted 23 interviews at the national level and 13 interview at the state level 6 in Lagos and 7 in Enugu (Table 1). Respondents were purposively selected from the stakeholders and were interviewed via face-to-face approach at respondents’ offices and via telephone calls both at the national and subnational level from organizations involved in the COVID-19 response to cover the 6 areas of the WHO Building block.

**Table 1:** Socio demographics of respondents

	National level	State level	Gender
**Policy maker**	12	8	14 males and 6 females
**Implementers**	5	2	2 males and 5 females
**Academia**	2	3	5 males
**Private consultant**	4		3 males and 1 female
**Total**	23	13	

These building blocks constitute the WHO health system framework, and they include service delivery, health workforce, health information systems, access to essential medicines, financing, and leadership and governance [15]. Service delivery implies services provided by the health sector and is measured in in terms of quality, access, safety and coverage while health workforce consists of human resources [15]. The health information system is a system that ensures timely and reliable information is produced, analysed, and disseminated while essential medicines entail procurement and supply of equitable, assured quality and cost-effective use of medical products [15]. Financing gives insight to healthcare funding, allocation of funds and purchase of healthcare goods and services while leadership and governance provides oversight, strategic policy frameworks, and accountability [15]. Ethical approval was obtained from the University of Nigeria Teaching Hospital Ethics Committee NHREC/05/01/2008B-FWA00002458-1RB00002323.

### Data management and analysis

Data extraction template consisting of the following variables (article name, lead organization/ author, publication details, source/URL, type of document and coverage, main purpose of document and building block discussed) and Standard operating procedure (SOP) was used for the document, media and journal reviews. The search was done by 2 persons to ensure consistency of findings and coverage of scope, A key informant interview (KII) guide was developed for the interviews with the stakeholders based on the research questions under the following headings (leadership and governance, healthcare financing, service delivery, health information management systems, human resource for health, medicines and technology community participation, partnership for health and research ) The interviews were conducted in pairs by 4 researchers trained in qualitative data collection. The guide was pretested among stakeholders at the national and state level in a state that was not selected for the study and corrected before use.

All interviews were audio recorded and transcribed verbatim. The interview transcripts were read repeatedly to identify words in the text that fell into the themes. The themes that were manually generated were entered into NVivo. The main themes were inputted as parent nodes while the sub themes were entered as child nodes. Transcripts were imported into the NVivo version 12 for coding and analysis was done thematically. Results were presented as quotes to supplement findings from the literature review about the engagement of the private and other sectors in the COVID-19 response.

## Findings

Our findings from the review of documents and interviews showed that the health system in responding to the COVID-19 pandemic, leaned towards a horizontal dimension of partnership that linked the health sector with different governmental sectors, and non-governmental sectors. All the components of the health system building block had a measure of partnership contributing to its accomplishments. For instance, a number of guidelines were produced, risk for the pandemic was widely disseminated, testing, and COVID-19 testing capacity was increased and these partnerships came in varied forms, ranging from, funding, technical assistance/ service support via donations to research and document development.

### Horizontal dimension of partnership in response to COVID-19 pandemic in Nigeria

Documents reviewed showed that in the course of managing the covid outbreak, a lot of partnerships were formed. These partnerships were formed by various government ministries, departments, and agencies (MDAs), Non-governmental organizations (NGOs) and Civil society Organizations (CSOs). From the preparatory stage to stem the viral outbreak, the country did not delegate the health system response to the health sector alone.

A private industry, and a private aviation firm, partnered with the Federal Airport Authority of Nigeria (FAAN) to safeguard the airports for workers and travellers as well as enlighten them on how to prevent contracting the virus [11]. The Federal Ministry of Health, in collaboration with a humanitarian aid organization, Alliance for International Medical Action (ALIMA) provided medical care for the vulnerable group, the internally displaced persons IDPs during the peak of the covid-19 pandemic [10]. The health sector also partnered with various other organizations like the Grassroot Initiative for Strengthening Community Resilience (GISCOR), the United Nations Office of the Coordination of Humanitarian Affairs (OCHA) community mobilization working group, community teams in the Water Sanitation and Hygiene (WASH) Sector, and community healthcare workers that were involved in the Polio eradication programs because of their networks and skills in community outreach [10].

Our respondents in the interviews, corroborated our findings from the literature review, stating other multilateral, bilateral agencies and countries that came in to partner with the government agencies.

*“We also have other bigger organizations like WHO, UNICEF, CDC, so we also have a smaller implementing partner. Before I will call the smaller organization, we also have GAVI, World Bank. World Bank is also part of the strong ones acting as donor. So, these are the major bigger organization. Then we have other implementing organizations which are country based, we have AFENET, even though AFENET is Africa but then African Field Epidemiology Network, that’s AFENET. We usually get their support through CDC”* (National level decision maker) Another national level decision maker also alluded to their organization partnering with other governmental and non-governmental organizations and he also stated *“Yes, we had a lot of partnerships. Initially we started with WHO and they have always been there for us. Also, we partnered with AFENET, Resource to save life, among others I can’t remember all of them now, but we have a lot of partners giving us their support”.* (National level Implementer)

### Partnership contributed to the accomplishment of the health system building blocks

Examining the country’s response to COVID-19 pandemic in the light of the six components of the health system building blocks showed elements of collaboration in every facet.

#### Leadership and governance

In terms of providing guidance documents and developing plans as a function of leadership, we also had partners participate and enhance the work at the national and state level, as stated by our respondents in the key informant interview.

*“And with the support of the World Bank, we were also developing the state incident action plans, and supporting them with funding to kick start preparedness within their state on all the identified gaps they have, from the risk assessment done”.* (National level decision maker)

Technical working groups are saddled with the responsibility of providing guidance with respect to implementing projects. Organizations other than the Federal Ministry of Health were part of this group providing a form of partnership in response to the pandemic both at the national and state level. A state level implementer alluded to this. He stated that the “*WHO, the NCDC and other NGOs had a lot of their staff that were active members of the technical working group”* (State level decision maker).

#### Healthcare financing

Most of the funding sources for combating the COVID-19 outbreak were from collaborations from private organizations, multilateral organizations, and even private individuals. The country, riddled with inadequate funding for healthcare could not have on its own been able to provide all the needed funds to fight the pandemic. Review of documents showed that as a way of garnering funds, the United Nations Nigeria launched a basket fund for COVID-19 which attracted quite a number of donors. The APM terminals donated N75 million to the basket fund whilst providing N25 million for promoting risk communication via radio and other social media platforms [16]. The Dangote foundation, donated N1.5 billion to the basket fund [17]. Within 6 months of the onset of the pandemic, the United Nations Nigeria had mobilized $61.3 million into the COVID-19 basket fund [18]. The coalition against COVID-19 (CACOVID), which is a group of private sector players raised about $55.7 million to support the government in containing the outbreak within the first two months following the diagnosis of the index case in Nigeria [18]. The Global Fund for HIV, Malaria and Tuberculosis re allocated $5.1 million for the country’s COVID-19 response, to support the purchase of gene-expert testing machines. Several other multilateral, bilateral and private organizations and foundations made cash donations towards containing the pandemic [18].

In the key informant interviews, one of our respondents corroborated the fact that CACOVID supported the government by providing funds for certain commodities he stated that *“there were a lot of donors, private sector donors who came together as a coalition called CACOVID, they were drawing their funds together, trying to procure items from the quantification and forecast we had done”* (National level decision maker).

Apart from the CACOVID other organizations and like foundations and even financial institutions provided funding support. According to some of our respondents, *“Yes so Bill Gates foundation is also funding some other organizations apart from also having some program officers that are supporting us technically at the national level”* (National level decision maker) *“I did say that Union bank for instance who came here with a cheque that was displayed to technical working group of two hundred and fifty thousand naira down as token and it was a huge sum of money”* (State level decision maker).

#### Service delivery

From the review of documents, we found that to enhance service delivery, 15,000 consultations were provided for Internally Displaced Persons (IDPs) by ALIMA and the federal ministry of health [10]. The effort of the health sector in testing for COVID-19 was boosted by a private health investment firm, ‘Flying Doctors’ which provided mobile sample collection booths in 8 states of the country that were worst hits of the pandemic. This increased the country’s daily testing to about 2500–3000 tests daily [19]. The NCDC co-opted private laboratories to participate in COVID-19 testing to increase the testing capacity of the nation [20].

In the key informant interviews, with respect to service delivery, our respondents mentioned that goods that enabled service delivery were provided by donors like the UNDP, *“We also were able to take things to primary healthcare within the state, that was supported by the UN, the UNDP supplied a lot of goods through UNICEF, for primary healthcare because those are the first point of care at the rural areas”*(National level decision maker). And also, the laboratory had support that enhanced its capacity to provide services. *“we now come to lab, we have a lot of guys from WHO like ‘DW’ is from WHO, supported laboratory, supported the lab pillar greatly, right, even in terms of establishing these molecular labs and all that to the country, okay, so, it is all about partnership”, (National level* decision maker)

#### Health workforce

There were also collaborations in building capacity of health workers and other workers relevant to the healthcare sector. Review of documents showed that ALIMA a new model humanitarian organization that connects national NGO’s and international research institutes trained healthcare workers and state burial teams on infection prevention and control measures [10].

From our respondents in the key informant interviews, partners like the US CDC partnered with the NCDC in building capacity of the health workforce. *“So, they have these partners of the US CDC, they’ve equally helped us in building the capacity of and capability of our frontline responders by introducing public health emergency management program or professional development program” (National level decision maker)*.

The partners did not only support in building the capacity of the health work force via trainings, they also brought in their own workforce to work with the health agencies in the country there by boosting the numerical strength of the health work force. *“Yeah, WHO helped out especially with emergency unit licensing, they helped us out, they came in they gave us professionals to work with, to look at our systems global best practices, our quality management systems. They helped, they came and helped”.* (National level implementer)

#### Health management information system (HMIS)

On health information management, the government partnered with communication companies according to our respondents in the key informant interviews. *“We are working with E-health, with the support of Bill and Melinda Gates foundation, and then the global fund, to finetune the logistic tool”* (National level decision maker).

On the other hand, a tech consulting firm told us that they conceptualized and developed the electronic management for data. *“Yes we supported the development of the electronic management for information data or immunization data and developing that system the one that you know when you get COVID19 vaccine…”* (National level private consultant) He stated that their organization wrote the concept note and provided the information technology expertise that developed the Electronic management data used by the NCDC for data transmission. *“Yes, we were part and parcel of conceptualizing, we actually wrote the first concept of that and then we worked through the process of developing it, designing it, reviewing the input, the whole work of architecture and we had the IT guys and we now focus on that and eventually you know got integrated into the system that we have now, so we played the role in supporting, in supporting the government to plan”.* (National level private consultant)

#### Medicines and technology

Review of documents showed that Nigeria did not produce any drug or vaccine for COVID-19 but with respect to other health commodities like personal protective equipment which came in short supply at the peak of the pandemic, about 2000 to 5000 coveralls and about 10, 000 nose masks were produced on a daily basis in Rivers State by 2 companies (Xirea Apparel and Buphalo Active Gear) that partnered to make this production possible [21].

### Forms of partnership

The partnerships came in varied forms, ranging from, funding, technical assistance/ service support via donations to research and document development.

Review of document showed that eHealth Africa, provided technical assistance to NCDC in the development of the COVID-19 online course on infection prevention and control (IPC) targeted at health workers but open to the public [22]. To support the services of the Federal Airport Authority of Nigeria (FAAN) and the airport managing team in safeguarding the airports, a private industry provided free hand sanitizers, wash gel, facial wipes and bar soaps for a period of one month [11]. Donation of medical supplies from Chinese businessman Mr. Jack Ma, helped to increase the country’s testing capacity for the virus [23]. The Manchester business school alumni donated medical supplies worth over N2 million to the NCDC and these included; face masks, anti-bacteria hand wash, ethyl rubbing spirit, hand gloves, hypo bleach, towels, cotton wool, methylated spirit, industrial mob bucket and clinical dustbin [24]. APM terminals also gave 10,000 test kits, 15 oxygen concentrators and various personal protective equipment (PPE), vaccines, Interagency Emergency Health Kit (IEHK)/ Post Exposure Prophylaxis (PEP) kits, and other vital health supplies, to the United nations office in Nigeria to support the Nigerian Government’s COVID-19 Response Plan and UNICEF’s work with children and families in Nigeria [25]. The Nigerian National Petroleum Company (NNPC)/ Shell Nigeria Exploratory and Production Company (SNEPCo) donated state of the art medical equipment and ambulance to Lagos State government to help in service delivery for COVID-19 in Lagos state [26].

Our respondents in the key informant interviews corroborated what we found in literature. some of our respondents stated how the private sector helped them. “*we had vehicles from the private sector, they donated a lot of vehicles to us for use during that period”* (National level decision maker) *“And Dangote and his group you know supported us at the early stage, because some donated ambulances that we are using for contact tracing and all that… Dangote foundations donated vehicles, and those ambulances, about six ambulances”*, (National level decision maker).

Our respondents also mentioned that the private sector was very helpful with the procurement of scarce medical products that was needed for testing and diagnosis of COVID-19 virus. The viral transport medium a laboratory medical product was one of such commodities that was provided by CACOVID *“In getting viral transport medium, at the point we couldn’t find it in this country, you know CACOVID now supported greatly by providing these viral transport medium and the extraction kit”.* (National level decision maker)

These organizations also partnered with the health sector to provide personal protective equipment for frontline health workers and other necessary consumables. Our respondent stated that *“Yes that’s what I’m saying private partnership, we have them. Some of them were donating PPEs, even international agencies were importing commodities for us, then all the consumables to be able to work”* (National level decision maker) Private individuals were not left out in the partnering with Nigeria’s health sector to provide needed consumables for combating the pandemic. One of our respondents at the state level stated that *“And of course people in diaspora they were sending in packages of you know consumables; the mask, the sanitizers, and the other things…”* (State level decision maker).

The presidential task force now the presidential steering committee and some international agencies made donations as well *“Then we also had the PTF do some procurements, we had international agencies make donation, they also took their list from the forecast, you know the quantification we had done, so agencies like UN were able to support us*” (National level decision maker).

Bilateral organizations and other agencies supported by providing commodities as well as funding. *“We had WHO supporting us both with personnel and goods and then some funding to support deployment. We also had other agencies, actually we had well over forty… twenty to forty agencies that supported the work we were doing”* (National level decision maker).

With regards to providing technical assistance, a national level decision maker listed a number of organizations that provided technical support for their activities during the COVID-19 pandemic. He mentioned SIDANI and IVAC stating as follows *“We also have SIDANI, SIDANI is a private organization, but actually, they are providing a lot of support in terms of technical support, as well as consulting for other bigger organization like GAVI. We also have IVAC, IVAC is International Vaccine Access Centre. So IVAC, they are also the technical support that we have and we are working with them”* (National level decision maker).

Other non-health commodities were also provided to enable a good working environment for the government organizations whose responsibility was to ensure that the pandemic was controlled *“we had other people that came and donated new generators, then solar and all that, you know, then the DG used that opportunity to equally renovate our public health lab and up till now there are some projects that are ongoing, you know, so WHO equally just donated some vehicles* (National level decision maker).

Telecommunication companies were not left out. They supported the health agencies with funds and airtime. *“We got massive support from telecommunications companies, both in terms of either funding money but also either giving us airtime and bandwidth you know we got support from variety of people in that order”* (National level decision maker).

*“MTN gave us some airtime most of these data providers they gave airtime”.* (National level decision maker)

## Discussion

The main focus of the bigger study, of which this paper is a part of, was to build collaborative learning in and across countries and regionally in West Africa and Central Africa (DRC) between formal reported evidence and the experiential learning from implementation in countries. In Nigeria, we learnt that the country’s health sector adopted a horizontal approach to partnership in responding to the COVID-19 pandemic. The health system building blocks on which the health sector functions had partnerships contributing to its accomplishments in fighting the pandemic and these partnerships were in the form of funding, service support, technical assistance etc.

The COVID-19 pandemic brought so many countries and organizations together, working as teams at different levels to halt the pandemic. The COVID-19 pandemic placed a huge burden on the Nigerian health system and exposed its weakness, but the Federal government of Nigeria involved different sectors to respond to the COVID-19 pandemic. Nigeria has witnessed several disease outbreaks but the most recent notable being the 2014 Ebola virus disease outbreak. Prior to the outbreak, the Nigerian government established the Nigerian Centre for Disease Control (NCDC) by bringing together the Epidemiology division, Avian Influenza Project and the Nigeria Field Epidemiology and Laboratory Training Program (NFELTP) of the ministry of health with the goal to prepare against disease outbreaks in the country [27]. The NCDC has partnerships with the WHO, US CDC, Economic Community of West African States (ECOWAS) Regional Centre for Disease Control, and recently, the University of Maryland Baltimore, the Robert Koch Institute, the Global Outbreak and Response Network and Public Health England, all focusing on specific aspects of its mandate [27]. One of the lessons learned from the Ebola success story was collaborations with the organized private sector which provided funds that was helpful in keeping operations on track before public funds was released [28]. As soon as the COVID-19 pandemic was declared a public health emergency of international concern, the federal government of Nigeria and the NCDC as the coordinating body for disease outbreaks in the country leveraged on its existing partnership while adopting new partnerships with other countries as well as organizations. This strengthened the components of the health systems building blocks to a great extent.

So many policy documents, regulations and guidelines were developed at the time. The production of these guidelines expressed the leadership and coordinating function of the participating organizations as these were possible through collaborations with private consultants who were funded by international foundations and bilateral organizations. The documents provided a guide to all the proceedings that took place at the peak of the pandemic and is still currently in use. The application of these guiding documents was very helpful in reducing the spread of the pandemic by limiting the number of new cases.

There was funding from different sources, the Nigerian government private individuals, bilateral and multilateral organizations, country governments and international private organizations. Of the total funds available to the country for fighting the pandemic, the funds from the public source was just about 10% of the funds garnered by the country while 90% was from private sector and the donor philanthropist community [18]. This shows the need to further explore other sources of funding for Nigeria’s healthcare. The funding support that Nigeria received to enable her respond to COVID-19 pandemic is similar to Afghanistan where the World Bank, provided US$ 100.4 million for strengthening coordination, surveillance, laboratories, provision of supplies, capacity building, Rapid Response Teams and continuation of essential health services [29].

There were collaborations in training staff, leading to a massive capacity building exercise for majority of health workers in the country and increased expertise of health workers in handling outbreaks. Similar collaboration was noted in United Arab Emirates (UAE) where a private organization Thumbay Group, partnered with the government health sector to provide training for all healthcare professional as well as conducting live drills [30]. This might have reduced the number of infections and deaths that would have occurred among health workers in the event that there was no training. We also noted collaborations in providing services like testing and consultations and developing and using technologies for managing health information and also with regards to vaccines. This translated to increased testing capacity of the nation with daily updates on the corona virus trend on the NCDC site with regards to number of new cases, number of treated and discharged cases and number of deaths. The above information being available has kept the country on its toes with the aim of reducing the number of cases to the barest minimum as well as serving as an encouragement to note that efforts put is yielding positive results. With regards to technology for managing health information, Ghana FELTP collaborated with the Department of Geography at the University of Ghana, to develop an interim real time data collection and reporting tool using “ArcGIS, preceding the roll out of the Surveillance Outbreak Response Management and Analysis system (SORMAS) by its health service division [31].

Another evidence of the result of the partnerships is the development of effective surveillance measures and monitoring approaches for the pandemic. This was as a result of the NCDC leveraging on expertise and innovations of partnerships that existed prior to the pandemic. Partnership in surveillance was also noted in Ghana where its health service division partnered with her field epidemiology training program, Ghana FELTP to enhance surveillance and contact tracing and this effort proved productive receiving credit for detecting 63% of all confirmed cases in Ghana [31]. The sudden surge of molecular laboratories from 3 at the onset of the pandemic to at least one in every state of the federation with a total 71 PCR laboratories in 35 states of the federation and FCT [32] also was a product of partnership, leveraging on the existing laboratories infrastructure established by partners for the HIV/AIDS and TB programs; the United States Government and The Global fund for HIV, TB and Malaria, that was re purposed to provide testing for the coronavirus [33]. Ghana’s testing capacity for COVID-19 virus was also increased via its partnership with National Public Health Reference Laboratory (NPHRL), National Veterinary Service Department (VSD) Laboratory and Centre for Scientific and Industrial Research (CSIR) Laboratory in Accra; Public Health Reference Laboratory, Tamale, the Tamale Teaching Hospital Laboratory, Navrongo Health Research Centre in the northern part of the country, the University of Health, Allied Sciences (UHAS) laboratory in the Volta Region and some unmentioned private laboratories that provided testing sites and this resulted to improved laboratory turnaround time for confirmation of suspected cases [31].

To highlight unpublished communal efforts in Nigeria, various organizations, NGOs and CSOs also supported the response to the pandemic by providing locally manufactured face masks, hand sanitizers and locally produced handwash equipment to the state ministry of health and some healthcare centers around them to reduce the high rate of health workers infection and encourage them. The Department of Community Medicine, University of Nigeria Teaching Hospital (UNTH) Ituku/Ozalla Enugu State, partnered with Baywood Foundation an NGO to produce hand sanitizers that were donated to the Enugu State Ministry of Health and the University of Nigeria Teaching Hospital. The mere knowledge that so many organizations were partnering with the health agencies motivated change in behaviour of health workers and led to improved access and care for those diagnosed with COVID-19.

In Africa as a continent, at the very beginning of the pandemic, the Africa centre for disease control (Africa CDC), partnered with the Southern Africa Centre for infectious Disease Surveillance (SACIDS) to form Africa Task Force on Coronavirus Preparedness and Response (AFTCOR) to strengthen existing monitoring systems, enhance laboratory diagnosis, improve surveillance which includes screening at points of entry, establish infection prevention and control practices in Healthcare facilities while ensuring clinical treatment of people infected with COVID-19 virus [34]. Four months into the global pandemic, the African Union Commission and the Africa CDC launched a Partnership to Accelerate COVID-19 Testing (PACT): Trace, Test and Track (CDC-T3) to strengthen the testing capacity across Africa [34]. In Ghana and Rwanda, a California-based firm, Zipline repurposed its high-speed drones that were used to deliver medical packages to clinics and hospitals to identifying COVID-19 hotspots and collecting samples [19]. The same was noted in Kenya, where the ministry of health collaborated with an organization that developed mSafari, a mobile phone based application to support contact tracing [19]. A cloth manufacturing company in Kenya switched its production line to producing surgical face masks and produced about 30,000 surgical masks per day, relieving the shortage of masks after the country made the use of masks compulsory. Along the same line, in the same country (Kenya) a 3D company also started producing face shields [34]. In Uganda, Kiira motors cooperation, an automobile manufacturer collaborated with Makerere school of public health to produce ventilators for critically ill patients, thus improving service delivery [34]. In Senegal, a research institute produced a very affordable test kit that yielded results in about 10 min [34]. These partnerships would not have been possible without high level political support. All the above findings imply that if these efforts recorded are sustained, tackling future outbreaks will be a lot easier.

One of the limitations of our study was that it did not establish the length of time these partnerships were to last and plans for future collaborations. We could not also compute the costs of the partnerships and detailing of the ways and means to ensure that they are sustainable and can be used for wide range of shocks to the health system.

In summary, in responding to the COVID-19 pandemic, partnerships provided funding support and technical assistance, supported in implementing and delivering of projects, facilitated research, data collection and analytics; and coordinated various response actions [35]. In addition, the collaboration adopted by the federal government strengthened all the pillars of the health system to a great extent. However, a lot more needs to be done towards sustaining these gains to strengthen the health system to become more resilient and be able to absorb the unprecedented shocks in future. It is recommended that partnerships be encouraged at all levels of healthcare in Nigeria and sustainability plans clearly defined. Implementing this recommendation will go a long way to help the country build a health system that is better able to adapt and respond to future challenges.

## Data Availability

The anonymized data is available but on request. You can contact the corresponding author, amchico2002@gmail.com.

## Abbreviations

AFENET: African Field Epidemiology Network
AFTCOR: African Task Force on Coronavirus Preparedness and Response
ALIMA: Alliance for International Medical Action
CACOVID: Coalition Against COVID − 19
CDC: Centre for Disease Control
CSO: Civil Society Organizations
DG: Director General
ECOWAS: Economic Community of West African States
FAAN: Federal Airport Authority of Nigeria
FMOH: Federal Ministry of Health
GAVI: Global Alliance for Vaccine Initiative
GISCOR: Grassroot Initiative for Strengthening Community Resilience
IDP: Internationally Displaced Persons
IEHK: Interagency Emergency Health Kit
LMIC: Low- and Middle-Income Countries
MDA: Ministries, Departments and Agencies
MTN: Mobile Telephone Network
NAFDAC: National Agency for Food and Drug Administration
NCDC: Nigerian Centre for Disease Control
NFELTP: Nigeria Field Epidemiology and Laboratory Training Program
NGO: Non- Governmental Organizations
NIMR: Nigerian Institute of Medical Research
NIPRD: National Institute for Pharmaceutical Research and Development
NNPC: Nigerian National Petroleum Company
NPHCDA: National Primary Health Care Development Agency
OCHA: Office of the Coordination of Humanitarian Affairs
ONSA: Office of the National Security Adviser
PACT: Partnership to accelerate COVID-19 Testing
PEP: Post Exposure Prophylaxis
PHE: Public Health England
PHI: Pro Health International
PPE: Personal Protective Equipment
PSC: Presidential Steering Committee
PTF: Presidential Task Force
SACIDS: Southern African Centre for Infectious Disease Surveillance
SNEPCO: Shell Nigeria Exploration and Petroleum Company
SORMAS: Surveillance Outbreak Response Management and Analysis System
UAE: United Arab Emirates
UN: United Nations
UNDP: United Nations Development Programme
UNICEF: United Nations Children Emergency Fund
UNTH: University of Nigeria Teaching Hospital
WASH: Water Sanitation and Hygiene
WHO: World Health Organization
